# Mechanisms and etiologies of thrombocytopenia in the intensive care unit: impact of extensive investigations

**DOI:** 10.1186/s13613-014-0024-x

**Published:** 2014-08-02

**Authors:** Nadiejda Antier, Jean-Pierre Quenot, Jean-Marc Doise, Robin Noel, Emmanuel Demaistre, Hervé Devilliers

**Affiliations:** 1 Service de réanimation polyvalente, Centre Hospitalier, Chalon sur Saone 71321, France; 2 Service de réanimation médicale, Centre Hospitalier Universitaire Le Bocage, 14, Rue Gaffarel, Dijon 21079, France; 3 Laboratoire d’hémostase, Centre Hospitalier Universitaire Le Bocage, Dijon 21079, France; 4 Service de médecine interne, Centre Hospitalier Universitaire Hôpital Général, Dijon 21079, France; 5 CIC-EC CHU de Dijon (INSERM CIE1), Dijon 21000, France

**Keywords:** Thrombocytopenia, Intensive care unit, Bone marrow aspiration

## Abstract

**Background:**

Thrombocytopenia is common in the intensive care unit. Potential mechanisms and etiologies behind this phenomenon are multiple and often entangled. We assessed the effect of a systematic approach, using routinely available tests, on the proportion of patients in whom the mechanism (primary objective) and etiology (secondary objective) of thrombocytopenia in a mixed intensive care unit (ICU) could be identified.

**Methods:**

Before-and-after study of all patients with thrombocytopenia was used. ‘Before’ group had no intervention. New standard operating procedures for thrombocytopenia management were introduced. In the ‘After’ group, bone marrow aspiration; determination of fibrinogen dosage, prothrombin time, factor V, D-dimers; assay of fibrin monomers, ferritin, triglycerides, lactic acid dehydrogenase, aspartate transaminase, alanine aminotransferase, vitamin B_12_, folates, reticulocytes, haptoglobin, and bilirubin were performed.

**Results:**

In the Before group (*n* = 20), the mechanism (central, peripheral, or mixed) was identified in 10 % versus 83% in After group (*n* = 23) (*p* < 0.001) (48% peripheral, 35% mixed). Before intervention, ≥1 etiology was identified in 15% versus 95.7% in the After group (*p* < 0.001).

**Conclusions:**

Systematic and extensive investigation using routine tests highlights the mechanisms and etiology of thrombocytopenia in most cases.

## Background

Thrombocytopenia is the most common hemostatic disorder in the intensive care unit (ICU) with a prevalence of around 50% [1],[2]. It has been shown to be associated with mortality through several factors, including platelet nadir, rate of thrombocytopenia at ICU admission, rate of thrombocytopenia on day 7, or lack of early recovery from thrombocytopenia [2]-[6]. Thrombocytopenia is defined as a platelet count of <150,000/mm^3^[2] and it is classified as severe if platelet count is <50,000/mm^3^, which is the case for 2% to 15% of patients [7].

Thrombocytopenia is often classified as being caused by a mechanism that is either central, peripheral, or mixed. This distinction is also important in order to apprehend the consequences of thrombocytopenia. Indeed, there is greater tendency towards bleeding when the cause is central. Conversely, there is an increased thrombotic risk in case of a peripheral mechanism, such as platelet activation, which can occur, for example, in the presence of disseminated intravascular coagulation (DIC), frequently encountered in the ICU [8],[9]. Platelet activation is also involved in cases of heparin-induced thrombocytopenia (HIT) [1], antiphospholipid syndrome (APS), and thrombotic microangiopathy (TMA), which, although rare, are nonetheless not to be neglected, as underlined in the recent guidelines for the management of thrombocytopenia in the ICU setting [10]. Sometimes, a central or peripheral mechanism can be highlighted but without any etiology being identified.

When a central mechanism is suspected, it is customary to perform bone marrow aspiration. Current guidelines [10] recommend that bone marrow aspiration (BMA) should not be systematic, but rather should only be considered in the absence of any obvious etiology of thrombocytopenia or if other cell lines are affected. However, thrombocytopenia in the ICU setting is often multifactorial [9], frequently involving both central and peripheral mechanisms. Thus, a peripheral etiology does not exclude central involvement. In addition, it is rare for thrombocytopenia to occur without concomitant anemia in the ICU, regardless of whether the origin is partially central or not. Therefore, in theory, BMA should be discussed for all thrombocytopenic patients.

To the best of our knowledge, no before-and-after study evaluating the impact of systematic investigation of the etiology of thrombocytopenia, including BMA, has ever been performed in the ICU setting to date.

In this context, our study aimed to determine the impact of a systematic approach using routinely available tests on the proportion of patients in whom the mechanism of thrombocytopenia in the ICU could be identified. The secondary objective was to determine the impact of this systematic approach on the proportion of patients in whom at least one etiology of thrombocytopenia could be identified.

## Methods

This before-and-after study was conducted in a 16-bed, mixed ICU in a non-academic hospital (Chalon sur Saône, France) between May and September 2012, in the context of a review of professional practices. From 2 May to 2 July 2012, the first 20 consecutive patients who met the inclusion criteria were included in the ‘Before group.’ From 22 July to 10 September 2012, after introduction of the new approach, the first 23 consecutive patients who met the inclusion criteria were included in the ‘After group.’ The study was approved by the local ethics committee (Comité de Protection des Personnes Est I, Dijon, France). The study was considered to be part of routine care, and the need for written informed consent was waived. All patients (or their next of kin in case of neurological deficit) were informed about the study, and information about the study was provided in written and oral format.

### Patients

All patients hospitalized in the ICU during the study period and with a confirmed diagnosis of thrombocytopenia, as defined by a platelet count of <150,000/mm^3^, were eligible for inclusion in the study.

### Study design

Using a before-and-after study design, all ICU patients with thrombocytopenia were managed according to the conditions of their time period and consecutively included. For patients in the ‘Before’ group presenting thrombocytopenia in the ICU, management was left at the physician's discretion. Physicians were free to perform as many or as few investigations as they felt necessary and were unaware that a study was taking place.

In July 2012, a review of professional practices was undertaken at the initiative of the Chief of the ICU, further to the publication of international guidelines for the management of thrombocytopenia in the ICU [10]. The new standard operating procedures for the management of thrombocytopenia were introduced in the ICU. The new procedures that all staff were instructed to follow (i.e., the ‘action plan’) are described below. The 4 T score [11]-[13] was calculated for all patients receiving heparin at the time of inclusion or in the week prior to inclusion. For patients with a 4 T score >3, testing for HIT antibodies was performed, as HIT is unlikely for a 4 T score of 0 to 3 [11]. The purpose of the new action plan, as well as the exact procedures it recommended, was explained to all physicians and nurses working in the ICU during interactive training sessions with a senior physician experienced in the management of ICU thrombocytopenia. The staff were aware that practices would be evaluated further to the introduction of new procedures but were not aware of the exact study objectives. Subsequently, for all ICU patients in the ‘After’ group, physicians were required to follow the action plan for all patients with a platelet count <150,000/mm^3^.

#### New action plan for the management of thrombocytopenia (defined as platelet count <150,000/mm^3^) as explained to the caregiving team

The following plasma assays must always be done within 24 h after discovery of thrombocytopenia (platelet count <150,000/mm^3^ plasma):

 Prothrombin time (PT) (patient PT/controls PT)

 Fibrinogen

 Factor V

 D-dimers

 Assay of fibrin monomers present or not in soluble complexes (Sta-Liatest FM)

 Ferritin

 Triglycerides

 Lactic acid dehydrogenase (LDH)

 Aspartate transaminase (AST)

 Alanine aminotransferase (ALT)

 Vitamin B_12_ and folates

In case of anemia, the same tests must be performed, as well as measurement of plasma level of reticulocytes, haptoglobin, and bilirubin.

In patients with no history of thrombocytopenia, OR in patients with a history of thrombocytopenia that has worsened, OR if another cell line is affected (anemia or leukopenia), bone marrow aspiration must be performed within 72 h of the discovery of thrombocytopenia. Bone marrow aspiration must systematically investigate the mechanism of thrombocytopenia (central, peripheral, mixed, undetermined) and criteria in favor of the presence of hemophagocytosis.

### Assessments

Baseline characteristics were recorded for all patients, namely, socio-demographic variables, Simplified Acute Physiology Score (SAPS) II, Sequential Organ Failure Assessment (SOFA) score, medical or surgical admission, platelet transfusion, and hemoglobin and leukocyte counts at the time of diagnosis of thrombocytopenia. We also recorded nadir platelet count, primary diagnosis, use of dialysis during thrombocytopenia, and presence of cirrhosis. The course of platelet count was recorded and classified as normalization in ICU (achieving a count ≥ 150,000/mm^3^), improvement without normalization in ICU, stability in ICU, or deterioration in ICU. Platelet counts were performed every day until normalization, discharge, or death (whichever occurred first), and at the physician's discretion thereafter. For each patient, in the ‘before’ and ‘after’ phases, all data from the tests recommended in the action plan were recorded when available.

For each patient, in the before and after phases, the mechanism of thrombocytopenia was recorded, if identified (central, peripheral, mixed, unidentified) as well as the underlying etiology(ies), if identified.

For DIC, the International Society for Thrombosis and Hemostasis (ISTH) score was used.

Dilution was considered as involved if massive transfusion or fluid infusion was performed, i.e., at least a blood volume in 24 h or an half of blood volume in 4 h.

During smears examination, the percentage of histiocytes was established by counting histiocytes in a sample of 200 nucleated cells. Phagocytosis of marrow cells was defined as the presence of intact cells (as opposed to cells debris), such as red cells, erythroblasts, platelets, or white cells, in the cytoplasm of macrophages. Hemophagocytosis was diagnosed when the percentage of histiocytes exceeded 2% of marrow nucleated cells and there was more than one hemophagocytic cell per field.

If at least one criterion in favor of a central mechanism was present, but none for a peripheral mechanism, thrombocytopenia was classified as central. If at least one criterion in favor of a peripheral mechanism was present, but none in favor of central mechanism, thrombocytopenia was classified as peripheral. If at least one peripheral criterion *and* one central criterion were observed, thrombocytopenia was classified as mixed. If no criteria for either central or peripheral mechanisms were observed, the mechanism was classified as unidentified.

### Statistical analysis

The primary endpoint was the percentage of patients in whom the underlying mechanism of thrombocytopenia was identified.

The secondary endpoint was the percentage of patients in whom at least one underlying etiology of thrombocytopenia was identified.

Based on the observed rate of mechanism determination of 10% patients in the Before group, we calculated that 17 patients in the After group would be sufficient to show a significant difference of 40%, with at least 50% patients in whom a mechanism could be identified, with a power of 0.8 and an alpha risk fixed at 0.05 in a bilateral situation.

Quantitative variables are described as median [interquartile range (IQR)] and qualitative variables as number (percentage). Patient characteristics were compared between the Before and After groups using the Mann-Whitney *U* test or Fisher's exact test, as appropriate.

Statistical analysis was performed using SAS version 9.3 (SAS Institute Inc., Cary, NC, USA).

## Results

Twenty patients were included in the before phase. ISTH criteria in search of arguments in favor of DIC were performed for 14 patients; detection of heparin-induced anti-platelet antibodies was performed for two patients; and one BMA was performed. No other investigations were performed in this group.

A total of 23 patients were included in the After group. Of these, 22 underwent BMA, of which 3 could not be interpreted as they were too diluted by excess blood. The rest of the tests were carried out according to the action plan. Six patients had a 4Ts score >3 and underwent HIT antibodies testing; all were negative.

The baseline characteristics of the study population are shown in Table 1. The population of the Before group was included between 2 May and 2 July 2012. During this period, 50 patients were admitted to the ICU, giving a prevalence of thrombocytopenia of 40%. In the After group, 23 patients were included between 22 July and 10 September 2012 out of 69 admissions during the same period, giving a prevalence of thrombocytopenia of 33.3 %. Twelve of the 20 patients (60%) in the Before group and 19 out of 23 patients (83%) in the After group had a platelet nadir lower than 80,000/mm^3^.

**Table 1:** Baseline characteristics of the two groups

	**Before group**	**After group**	***p*** **value**
	**(*n*** **= 20)**	**(*n*** **= 23)**	
Age, median [IQR], years	63.5 [59;67]	69 [65; 84]	0.09
Female sex (%)	10 (50)	13 (56)	0.76
Nadir platelet count, median [IQR]/mm^3^	84,000 [52,000; 98,000]	34,000 [17,000; 76,000]	0.09
Hemofiltration (%)	8 (40)	14 (61)	0.22
Cirrhosis (%)	2 (10)	4 (17)	0.67
Sepsis (%)	20 (100)	17 (74)	0.23
Death in ICU (%)	6 (30)	15 (65)	0.03
SAPS II, median [IQR]	52.5 [41; 63]	72.5 [55; 78]	0.2
SOFA score, median [IQR]	11.5 [7; 15]	17 [9; 18]	0.1
Thrombocytopenia at admission to ICU (%)	10 (50%)	16 (70%)	0.23
Time from ICU admission to occurrence of thrombocytopenia, median [IQR], days	1.5 [1; 2]	1 [1; 2]	NS
Platelet transfusion, *n* (%)	2 (10%)	1 (4.3%)	0.59
Medical admissions	10 (50%)	18 (78%)	0.06

IQR, interquartile range; ICU, intensive care unit; NS, non-significant.

### Mechanisms

In the Before group, the mechanism of thrombocytopenia (central vs peripheral) could not be determined in 90% of patients, and in the remaining 10%, mixed thrombocytopenia was retained.

The proportion of patients with a formally diagnosed mechanism of thrombocytopenia increased from 10% to 83% between the two periods (*p* < 0. 001).

In the After group, a peripheral mechanism (without a central mechanism associated) was identified in 11/23 patients (48%) and mixed origin in 8/23 (35%).

### Etiologies

In the Before group, three patients (15%) had at least one identified etiology. Hemophagocytosis was retained as a cause of thrombocytopenia for one (5%) patient and DIC for two (10%) patients. No specific treatment was administered after identification of these phenomenons. No heparin-induced thrombocytopenia was diagnosed.

In the After group, 21/23 (91.3%) patients had at least one identified etiology. The percentage of etiology determination increased by 76% between the two periods (*p* < 0. 001). Etiologies were not mutually exclusive, and 9/23 (39%) patients had more than one etiology (two etiologies for 4 patients, three for 3 patients, four for 2 patients). The etiologies identified in the After group are described in Table 2. No heparin-induced thrombocytopenia was diagnosed. One patient with intra-medullary hemophagocytosis received corticosteroids and intravenous immunoglobulins. Two others received folate supplementation after deficiency was detected. There was no other specific therapeutic impact of the investigations in this group.

**Table 2:** Etiologies in the After group

**Mechanism**	**Central (*n*** **= 8)**	**Peripheral (*n*** **= 19)**
Etiologies, *n* (%)	Hemophagocytosis, 5 (21.7)	DIC, 5 (21.7)
	Folate deficiency, 2 (8.7)	Dilution, 2 (8.7)
	Myelodysplasia, 1 (4.3)	Hemofiltration, 6 (26)
		Hypersplenism, 1 (4.3)
		Other, 12 (52.2)

DIC, disseminated intravascular coagulation; Other, other peripheral causes (more specific etiology not provable).

### Course of platelet count and prognosis

Normalization of platelet count occurred for 16 patients (80%) in the Before group and for 9 patients (39%) in the After group (*p* = 0.01). The median [IQR] number of days required to achieve normalization was 7.5 [5; 8.5] days in the Before group and 5.5 [2; 7] days in the After group (*p* = 0.55). The relationship between the course of platelet count and prognosis in the After group is shown in Table 3.

**Table 3:** Course of platelet count and prognosis in the After group

	**Survivors*n*** **= 8**	**Non-survivors*n*** **= 15**	***p*** **value**
No improvement	2 (25%)	9 (60%)	0.19
Early improvement (≤day 3)	6 (75%)	3 (20%)	0.02
Late improvement (>day 3)	2 (25%)	3 (20%)	NS
Normalization	5 (62.5%)	4 (26.6%)	0.18

NS, non-significant.

### Platelet nadir and prognosis

Nadir platelet count was below 75,000/mm^3^ in all patients who died in the ICU. Three patients had a nadir below 5,000/mm^3^ and these three died, but we also observed very low nadir among survivors (17,000/mm^3^ minimum).

### Bone marrow aspiration

In the Before group, one (5%) BMA was performed. In 11 patients in the After group, a central mechanism was ruled out after BMA. Five (26.3%) specimens showed significant hemophagocytosis. All five of these patients had sepsis, and four were in shock with multiorgan failure. One other specimen showed myelodysplasia in a context of documented myelodysplastic syndrome. No bone marrow infiltration by abnormal cells was observed. One serious side effect occurred in the After group further to sternal BMA, namely, compressive pneumothorax in a context of irradiated sternum.

### Hemophagocytosis and hemophagocytic lymphohistiocytosis

We compared rates of classic biological parameters usually associated with hemophagocytic lymphohistiocytosis (HLH) and outcomes between the patients who showed hemophagocytosis on bone marrow aspirate and those who did not. Death in ICU, platelet count normalization, ferritin, aspartate transaminase, alanine aminotransferase, triglycerides, lactic acid, prothrombin time, fibrinogen, factor V, and leukopenia were not significantly different between these two groups. Only nadir platelet count was significantly lower in the intra-medullary hemophagocytosis group with a median (IQR) at 7,000/mm^3^ (5,000; 37,500) versus 65,000/mm^3^ (18,000; 78,000) in the other group (*p* = 0.03).

## Discussion

Our study demonstrated that systematic, extensive investigations, even if only using tests that are available in routine practice, make it possible to identify in the majority of cases whether thrombocytopenia in ICU patients is due to central, peripheral, or mixed mechanisms (83% versus 10% when interventions were not systematic). Moreover, routine investigation also makes it possible to identify at least one etiology for a large majority of patients. The systematic biological work-up that was performed in the After group in our study was established based on the main etiologies usually described in the ICU and also on the basis of the recent guidelines for the management of thrombocytopenia in the ICU setting [10].

Not surprisingly, a peripheral mechanism is involved in thrombocytopenia in most cases and is the sole cause in almost 50%. Conversely, we observed no case caused by a central mechanism alone, highlighting the importance of peripheral causes in ICU patients, as previously reported [9]. Our study confirms that this is valid even when extensive investigations are performed to identify the causal factors in all patients with thrombocytopenia. The prevalence of thrombocytopenia in our study was comparable to rates previously reported in the ICU setting [1],[2].

Our study is one of the few studies published to date that investigates the causes of thrombocytopenia using a rigorous etiological approach, with systematic implementation of BMA. It is the first to compare this systematic and extensive approach with the more classical approach. Indeed, in the prospective study by Stephan et al. [9] in surgical ICU patients, BMA was only performed when other cell lines were affected, or in the absence of other obvious causes considered, and, in the end, was only performed in 12 of the 52 patients included. In the study by Thiolliere et al. [14], the authors used a rigorous methodology with the extensive investigations, but they considered sepsis as one of the possible etiologies while we did not consider sepsis as an etiology in our study. Indeed, a lot of etiologies can be involved in sepsis (DIC, hemophagocytosis, hypoplasia induced by inflammatory mediators, immune destruction, hypersplenism). We did try to identify two etiologies classically involved in thrombocytopenia during sepsis, namely DIC and intra-medullary hemophagocytosis.

We noted one case with undetermined etiology in the After group of our study. In the study by Thiolliere et al. [14], there were six patients (2%) with undetermined etiology. Indeed, some etiologies are difficult, if not impossible, to establish because there is no specific test and multiple etiologies may be associated. This is the case, for example, for dilution, hypersplenism, and septic consumption. Drug-induced thrombocytopenia is also hard to affirm. Lim et al. [8] estimated its frequency at 18.8%, but in practice, this remains hard to confirm, since the diagnostic criteria are based on an improvement in platelet count when the drug is stopped. This often occurs anyway when sepsis is resolved. As regards DIC, no gold standard exists for the diagnosis of this condition and is also difficult to affirm especially in liver failure. We used the ISTH score in our study, in which recent guidelines recommend for the diagnosis of DIC [15]. To be fully comprehensive in highlighting possible etiologies for thrombocytopenia, a routine test to detect anti-platelet antibodies is lacking. However, it would likely be of little use in clinical practice, since there are no specific management approaches to be implemented when anti-platelet antibodies are detected. For hemophagocytosis, there is no consensus to date regarding a cytologic definition. The diagnostic criteria we used here were those used in a study by Stephan et al. [16] and Strauss et al. [2], also performed in the ICU setting.

According to these criteria, five patients' BMA revealed the presence of hemophagocytosis, but there is no consensus regarding its management in the ICU, especially in case of septic shock, apart from symptomatic and antibiotic treatment. Some authors advocate immunosuppressant therapy (corticosteroids and etoposide) for HLH, while others are more reluctant [17]-[22]. Indeed, in most cases, hemophagocytic syndrome in septic patients is reactive and does not require specific therapies, since the treatment of the sepsis alone usually leads to the resolution of hemophagocytosis. HLH is also cited in the guidelines as a diagnosis that must be envisaged, because it requires specific investigations and initiation of urgent targeted treatments. Moreover, classifying hemophagocytosis as an etiology with a central mechanism is open to debate. Indeed, during HLH, bone marrow richness is usually (but not always) normal or increased [23], and hemophagocytosis is often both intra- and extra-medullary. In our study, however, we only documented intramedullary hemophagocytosis and considered that any phenomenon occurring in the bone marrow was associated with a central mechanism.

Bone marrow aspiration is an invasive diagnostic test and is reportedly associated with a rate of serious adverse reactions of <1% [24]-[28]. It is not recommended systematically by current guidelines. The potential morbidity associated with this technique underlines the need to investigate its usefulness. Baughman et al. [29] reported that BMA did not yield relevant information that would have changed the management of the patient in any of the cases in their study. In contrast, Stephan et al. [9] reported that BMA made it possible to obtain a diagnosis in 10 cases out of 12. Thiollière et al. [14] reported that BMA provided new information in 22% of the cases and was likely to have an impact on patient management in 11%. In our study, BMA had an therapeutic impact for one patient who received a specific therapy for HLH. It should be noted, however, that the only 10% of the patients with an identified mechanism in the Before group did undergo BMA. Conversely, in the After group, the 83% of patients in whom the mechanism could be identified had BMA, and the 17% with no identified mechanism did not have an interpretable BMA. Given that the mechanism of thrombocytopenia is crucial to decision-making as regards transfusion and anticoagulation, we purport that this strategy has a major impact on patient management. Indeed, the greatest risk incurred by central thrombocytopenia is bleeding, while peripheral thrombocytopenia tends to be due to thrombogenic phenomena through platelet activation mechanisms. Therefore, anticoagulation and platelet transfusion may be either recommended or not recommended, depending on the type of thrombocytopenia involved and its underlying mechanism. Currently, there is no other test available to distinguish between central and peripheral mechanisms. Immature platelet fraction assessment could be useful to avoid BMA in some cases but has never yet been tested in the ICU setting [30]-[35]. Other studies are necessary to determine the feasibility and the impact of such a systematic approach on morbidity and mortality.

Regarding the specific therapeutic impact of the investigations performed in the context of the Action plan, which concerned only three patients in the After group, we worked on the hypothesis that the thrombocytopenia could be the result of a rare pathology, but for which an established specific treatment exists, such as vitamin deficiency, HIT, APS, or TMA. For example, although HIT occurs in less than 1% of patients in the ICU setting [36], the potential gravity justifies systematically searching for this diagnosis and the same is valid for APS and TMA.

Our study has several limitations that need to be acknowledged. Firstly, it was performed at a single center with a small number of patients, and the results may not be generalizable to other institutions. Second, the use of a before-after study design precludes any firm conclusion regarding causality. Although the design of this study does not allow comparison of outcomes between the two populations, it appears nonetheless that the After population had a higher mortality rate than the Before group. SAPS II and SOFA score did not differ significantly between periods, but this could be due to the small number of patients included. We noted a borderline significant trend towards more medical than surgical admissions in the After group, which might partially explain the higher mortality in this group. However, it is unlikely that heterogeneity in the severity of disease between the groups would impact on the main objective of this study, considering that the platelet nadir did not differ significantly.

## Conclusions

Our study shows that systematic and extensive investigations using routinely available tests make it possible to distinguish between central, peripheral, and mixed mechanisms in a large majority of thrombocytopenic patients. It also makes it possible to identify at least one etiology of thrombocytopenia in most cases.

## Abbreviations

APS: Antiphospholipid syndrome

BMA: Bone marrow aspiration

DIC: Disseminated intravascular coagulation

HIT: Heparin-induced thrombocytopenia

HLH: Hemophagocytic lymphohistiocytosis

ICU: Intensive Care Unit

IQR: Interquartile range

ISTH: International Society for Thrombosis and Hemostasis

SAPS II: Simplified Acute Physiology Score II

SOFA: Sequential Organ Failure Assessment

TMA: Thrombotic microangiopathy

## Competing interests

The authors declare that they have no competing interests.

## Authors’ contributions

NA, JMD, HD, and JPQ are involved in the study conception and design. NA, JMD, and RN are responsible for the data collection. NA, JPQ, HD also performed the statistical analysis. All authors interpreted the data. NA and JPQ drafted the final manuscript. All authors read and approved the final manuscript.
